# Supplementary material for: A Microbial Co-Culturing System for Producing Cellulose-Hyaluronic Acid Composites
Source: Microorganisms. 2023 Jun 5;11(6):1504. doi: 10.3390/microorganisms11061504 (PMC10305311; doi:10.3390/microorganisms11061504)
Supplement: Supplementary file 1 [file microorganisms-11-01504-s001.zip › microorganisms-2418503-supplementary.pdf]

A microbial co-culturing system for producing cellulose-hyaluronic acid composites

Marcello Brugnoli[1], Ilaria Mazzini[1], Salvatore La China[1], Luciana De Vero[1] and Maria Gullo [1,2,*]

[1] Unimore Microbial Culture Collection Laboratory, Department of Life Sciences, University of Modena and Reggio Emilia, Reggio Emilia, Italy; marcello.brugnoli@unimore.it (M.B); ilaria.mazzini@unimore.it (I.M) salvatore.lachina@unimore.it (S.L.C); luciana.devero@unimore.it (L.D.V.); maria.gullo@unimore.it (M.G)

[2] NBFC, National Biodiversity Future Center, Palermo 90133, Italy

* Correspondence: mari.gullo@unimore.it

Table S1: Average fiber diameter of pure BC produced by K1G4 and K2G46, and of BC-HA composites produced by UMCC 2947- UMCC 2535 (C1), UMCC 2947- UMCC 2496 (C2), UMCC 3071- UMCC 2535 (C3), and UMCC 3071- UMCC 2496 (C4).

	**K1G4**	**C1**	**C2**	**K2G46**	**C3**	**C4**
Fiber diameter	$60.6^{c} \pm 17.7$	$82.1^{b} \pm 32.9$	$77.8^{b} \pm 23.4$	$62.5^{c} \pm 25.0$	$110.2^{a} \pm 47.2$	$109.6^{a} \pm 30.5$

Data are expressed as means ± standard deviations. Different letters indicate statistical differences within the same row at $p \leq 0.05$.
